# Reorganization of sea urchin gene regulatory networks at least 268 million years ago as revealed by oldest fossil cidaroid echinoid

**DOI:** 10.1038/srep15541

**Published:** 2015-10-21

**Authors:** Jeffrey R. Thompson, Elizabeth Petsios, Eric H. Davidson, Eric M. Erkenbrack, Feng Gao, David J. Bottjer

**Affiliations:** 1 Department of Earth Sciences, University of Southern California, Los Angeles, California 90089-0740; 2 Division of Biology, California Institute of Technology, Pasadena, California, 91125.

## Abstract

Echinoids, or sea urchins, are rare in the Palaeozoic fossil record, and thus the details regarding the early diversification of crown group echinoids are unclear. Here we report on the earliest probable crown group echinoid from the fossil record, recovered from Permian (Roadian-Capitanian) rocks of west Texas, which has important implications for the timing of the divergence of crown group echinoids. The presence of apophyses and rigidly sutured interambulacral areas with two columns of plates indicates this species is a cidaroid echinoid. The species, *Eotiaris guadalupensis,* n. sp. is therefore the earliest stem group cidaroid. The occurrence of this species in Roadian strata pushes back the divergence of cidaroids and euechinoids, the clades that comprise all living echinoids, to at least 268.8 Ma, ten million years older than the previously oldest known cidaroid. Furthermore, the genomic regulation of development in echinoids is amongst the best known, and this new species informs the timing of large-scale reorganization in echinoid gene regulatory networks that occurred at the cidaroid-euechinoid divergence, indicating that these changes took place by the Roadian stage of the Permian.

Living echinoids, members of the phylum echinodermata, belong to either the Cidaroidea or Euechinoidea, and these two subclasses comprise the crown group echinoids^1^. The differential morphological diversity of these two subclasses is striking. Since their emergence, euechinoids have diversified extensively from the bauplan of their earliest representatives^2^. For example, some euechinoid clades, such as the Irregularia, which includes heart urchins, have secondarily gained anterior-posterior bilateral symmetry^1^,^2^. In contrast, cidaroids have never strayed far from the body plan of the earliest cidaroids. Neither the euechinoids nor cidaroids, however, are known to be more basal than the other, and the Paleozoic Archaeocidaridae, from which the euechinoids and cidaroids likely evolved, display synplesiomorphic characters of both^1^,^3^.

The genetic and molecular developmental assembly of the echinoid bauplan is amongst the best understood for any taxon^4^,^5^,^6^ and a large-scale reorganization of echinoid gene regulatory networks (GRNs) underlay the initial divergence of cidaroids and euechinoids^7^. Developmentally, cidaroids and euechinoids are also strikingly different. Cidaroid embryos possess a variable number of micromeres, whereas those of euechinoids possess a characteristic four^8^,^9^. Embryonic cidaroids also lack primary mesenchyme cells^9^, from which the larval skeleton arises in euechinoids^10^,^11^. Recent work has begun to explore the genomic underpinning responsible for these morphological differences in early development^7^. One of the key differences between the euechinoid and cidaroid skeletogenic GRNs is the likely absence from the genome of the *pmar1* first repressor in the double negative gate^6^ of cidaroids^7^,^12^. The double negative gate is a regulatory circuit wiring design that is key to the specification of skeletogenic mesenchyme in euechinoids and the use of which in skeletogenesis is probably peculiar to this clade.

Echinoids are important and common constituents of modern ecosystems^13^,^14^,^15^. Though they have a diverse and storied history ranging back more than 400 myr to the Ordovician^16^, echinoids do not become abundant in the fossil record until 200 myr later in the Mesozoic^1^,^2^. Echinoids radiated in the Mesozoic after undergoing a bottleneck at the Permo-Triassic mass extinction (252 Ma) where they experienced a severe reduction in diversity^17^,^18^. The euechinoidea and cidaroidea clearly diverged before this mass extinction at the end of the Permian^19^, though the details of the timing of this divergence are not well constrained due to the rarity of echinoids in Palaeozoic strata. Apart from disarticulated spines, echinoids in the Palaeozoic are exceedingly rare. Most Palaeozoic echinoids had poor preservation potential compared to post-Palaeozoic forms, with many clades displaying imbricate, overlapping, plating which presumably lacked stereomic interlocking^20^,^21^. Because of this non-rigid test plating, Palaeozoic echinoids presumably disarticulated rapidly following their death, and thus well-preserved specimens in the Palaeozoic are usually limited to Lagerstätte deposits^22^. The stem-group cidaroid herein described from the Guadalupian of Texas, *Eotiaris guadalupensis* n. sp., is the earliest putative crown group echinoid known in the fossil record, and as such, provides new insight to the timing of the divergence of the euechinoids and cidaroids, which must have preceded it, and the associated morphologic and developmental gene regulatory changes that are the basis for this divergence.

## Stratigraphy and Geologic Setting

All new specimens of *Eotiaris guadalupensis* n. sp. are known from the Lamar Member of the Bell Canyon Formation in the Guadalupe Mountains of west Texas (Supplementary Fig. S1). Specimens described by Kier^23^,^24^ are from the Word and Road Canyon Formations of the Glass Mountains of west Texas (Supplementary Fig. S1). The Lamar Limestone is Lower Capitanian, about 264–263 Ma and the Road Canyon Formation is, at its youngest, 268.8 Ma. Stratigraphy and geologic setting is herein treated for only newly described material and detailed stratigraphic and locality information are in supplementary information.

## Systematic Palaeontology

Class Echinoidea Leske, 1778

Subclass Cidaroidea Smith, 1984

Family Miocidaridae, Durham and Melville, 1957

*Type genus—Miocidaris* Döderlein, 1887

*Other genera—Eotiaris* Lambert, 1900*, Couvelardicidaris* Vadet, 1991, *Procidaris* Pomel, 1883

Genus *Eotiaris* Lambert, 1899

*Type species—Cidaris keyserlingi* Geinitz, 1848, from the Wuchiapingian Zechstein of Germany and England.

*Diagnosis—*Miocidarid with small test. Interambulacral plates imbricate adapically. Areoles confluent only at and below ambitus. Spines with spinules, clavate to bulbous.

*Occurrence—*Upper Permian of Germany, the U.K. and now Guadalupian of Texas, USA.

*Remarks—*The name *Eotiaris* is used instead of *Miocidaris* as the type material of the type species of *Miocidaris* is indeterminate. The name *Miocidaris* was first used by Döderlein^25^ who failed to explicitly name a type species for the genus. Bather^26^ then designated *Cidaris klipsteini* Desor, 1855 as the type species, renaming it *Miocidaris cassiani* since it was preoccupied by *C. klipsteini* Agassiz & Desor, 1847. *M. cassiani*, itself, however, is a junior objective synonym of *C. ampla* Desor, 1858, a name proposed by Desor in the Addendum to his synopsis when he realized that his *C. klipsteini* was preoccupied^27^. Bather’s lectotype^26^ consists of just fragmentary interambulacral plates^28^, which are indeterminate at the generic level, and are best left restricted to the type material. Geinitz^29^ and King^30^ described the taxa *Cidaris keyserlingi* Geinitz and *Cidaris verneuiliana* King from the Wuchiapingian of the UK and Germany. King^31^ then placed *Cidaris verneuiliana* into *Archaeocidaris*, however this taxon does not have the four interambulacral columns that characterize *Archaeocidaris*. Desor^32^ furthermore placed *Cidaris keyserlingi* into *Eocidaris* however, this genus is strictly indeterminate, being based solely off of disarticulated interambulacral plates. Lambert then proposed the name *Eotiaris keyserlingi* for the material described by Geinitz. We follow Bather^26^ and Smith and Hollingworth^19^ in synonymizing *Cidaris keyserlingi* Geinitz and *Cidaris verneuiliana* King. Because the type of *Miocidaris*, however, is indeterminate, the genus should only be restricted to the type species, *Miocidaris ampla* (Desor) from the Carnian St. Cassian beds. Lambert’s name *Eotiaris* is thus the oldest available name for the material described by Geinitz and King and is used herein.

*Eotiaris guadalupensis* Thompson n. sp.

1959 Spine Kier 1958a p. 889 Plate 114 Fig. 3.

1965 *Miocidaris* sp. Kier 1965 p. 456.

*Type—*Holotype is USNM 610600, paratypes are USNM 610601-610605.

*Diagnosis—Eotiaris* with straight, clavate and bulbous spines covered in numerous spinules arranged helically around the shaft.

*Derivation of name—guadalupensis* from the Guadalupe Mountains of west Texas, from where the type material was collected.

**Figure 1:**
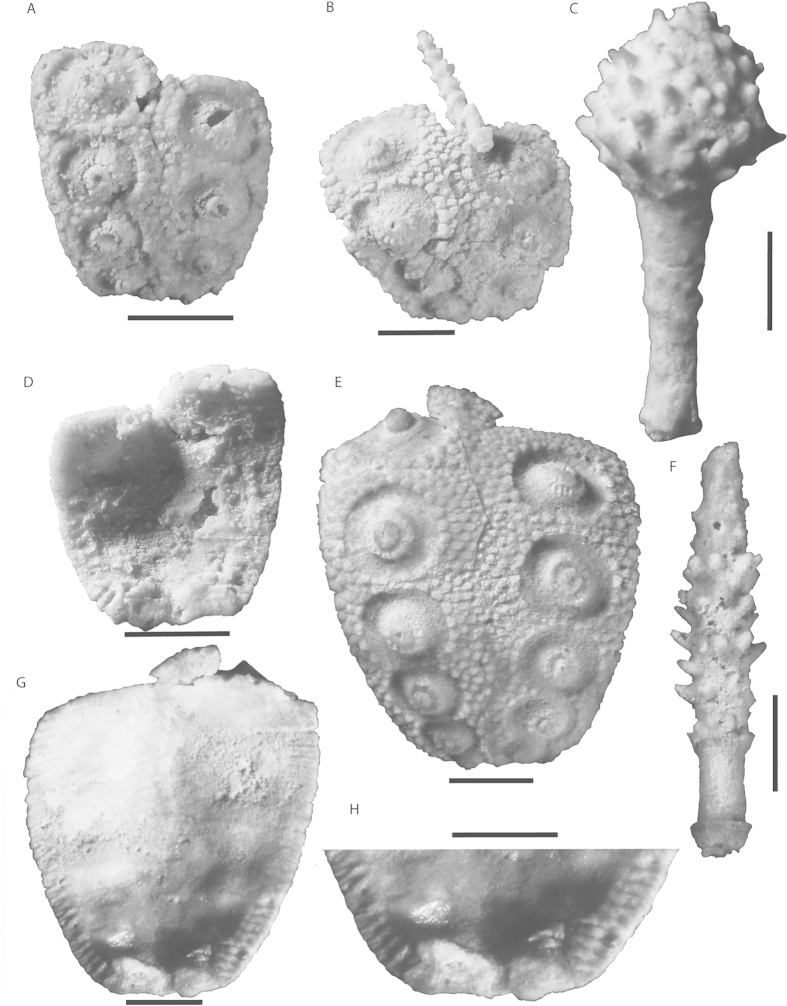
*Eotiaris guadalupensis* n. sp. (**A**) Paratype USNM 610604. Interambulacral area fragment first mentioned in Kier^24^ from Roadian of the Glass mountains. Note two column interambulacral area structure indicative of crown group echinoids. (**B**) Holotype USNM 610600. Interambulacral area fragment and associated spine. Note crenulate tubercles. (**C**) USNM 610605a. displaying clavate, bulbous spine morphology. (**D**) Paratype USNM 610604. Internal view of interambulacral fragment showing apophyses at adoral end. (**E**) Paratype USNM 610601. Interambulacral fragment of larger specimen. Note at least six plates in ambulacral columns and crenulate tubercles with sunken areoles. Plates rigid at least below adapical plates. **(F**) Paratype USNM 610605b. Spine displaying less clavate morphotype and spinules. (**G**) Internal view of interambulacral area of paratype USNM 610602. Note apophyses, which identify this species as a cidaroid, and denticulate adambulacral plate margin indicative of beveling. (**H**) Close up of apophyses of USNM 610602. All scale bars represent 2.5 mm.

*Description—*Test regular and small, known only from disarticulated interambulacral columns. Columns range in width from 4.2 mm to 9.3 mm (Fig. 1A,B,D,E,G). Modern cidaroids have an interambulacral ambital width about 45% of their test diameter^19^, thus estimated *E. guadalupensis* test diameters about 9.4 mm to 20.6 mm. Apical system unknown, and adapical interambulacral plates are not preserved articulated to the interambulacral columns of the test. Adapical interambulacral plates likely imbricate whereas ambital and adoral interambulacral plates rigidly sutured (Fig. 1E). Peristomial plates unknown, however apophyses are present on most oral interambulacral plates (Fig. 1G,H). No buccal notches present.

Lantern and teeth unknown. Ambulacra unknown, although likely beveling under interambulacral plates as interior adradial interambulacral plate edges are denticulate.

Interambulacral plating arranged into two rows. First four to six plates usually rigidly sutured with more adapical plates disarticulated (Fig. 1D,E). Plates pentagonal, about 1.3 to 1.6 times as wide as high. Primary tubercles large, sunken, and confluent below ambitus (Fig. 1A,E). Areoles at ambitus on specimen USNM 610601 about 2.6 mm wide and 2.6 mm high. Boss crenulate with mamellons undercut and perforate. At ambitus, one row of secondary tubercles on each plate separates tubercles. Above ambitus, multiple rows of secondary tubercles separate ambitus on large specimens. On large specimens, about four rows of secondary tubercles between the edge of each tubercle and the perradial suture at ambitus (Fig. 1E). About three rows of secondary tubercles between primary tubercles and adradial suture at ambitus. Adorally, this is reduced to two rows and eventually one row on the most adoral plates. On smaller specimens, the number of secondary tubercles arranged laterally to the primary tubercles are reduced to one. Interior of interambulacral plates slightly concave with seven or eight denticles per plate at ambitus.

Spines ranging in morphology from straight (Fig. 1F) to clavate to bulbous (Fig. 1C). Proximal fourth to third of spine shaft smooth, ending in diagonally oriented ridge, which contains the first row of spinules. Spinules oriented diagonally, along this raised ridge with more distal rows parallel to first row. Spine morphology variable, with some maintaining constant width and others tapering distally. Others ending in large clavate bulb covered in spinules. It is likely that spines varied aborally to orally, as is present in some archaeocidarids^22^ and recent cidaroids such as *Eucidaris clavata*^33^. Although this variability exists, all spines of differing morphologies contain diagonally oriented ridge bearing first row of spinules. Acetabulum of spine bearing perforation and faint crenulations. A single non-clavate spine is found associated with an interambulacral fragment which is 5.0 mm in length (Fig. 1B). The interambulacral fragment is 7.6 mm wide indicating a probable test diameter of 16.8 mm. This would indicate that the spines were likely less wide than the diameter of the test. Spines have a prominent milled ring proximally. Bulbous spines hollow distally in bulb and non-bulbous spines hollow distally. Secondary spines and pedicellariae unknown.

**Figure 2:**
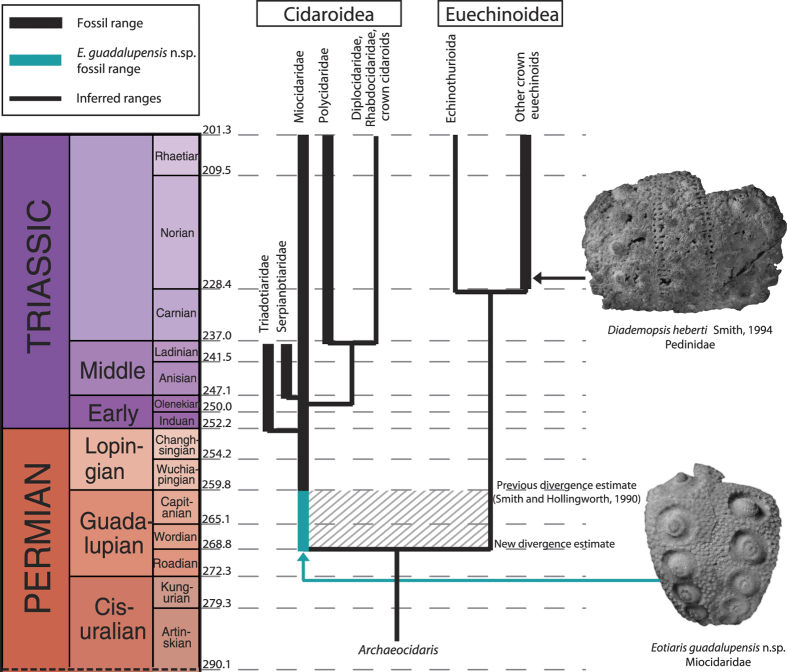
New divergence date of the divergence of cidaroid and euechinoid clades based on the Roadian occurrence of *Eotiaris guadalupensis* n. sp. Thick lines represent fossil range and thin lines represent inferred range based on phylogenetic relationships. The establishment of *E. guadalupensis* as the oldest known cidaroid in the fossil record also extends the inferred range of euechinoids, as the oldest known euechinoids, *Diademopsis herberti,* and *Hemipedina hudsoni* are first found in the fossil record in the Norian, 40 Ma years later. Phylogenetic relationships are from Kroh and Smith^1^ and Kroh^35^ modified with information regarding phylogenetic placement of *E. guadalupensis* from Supplementary Figure S2.

*Remarks—*This taxon has been mentioned previously by Kier^23^,^24^ from the Roadian and Wordian of west Texas, albeit as a single disarticulated interambulacral area and as misidentified cidarid secondary spines respectively. The inclusion of more material, and the association of the spines with the test of this species allow for a more thorough description herein. All new specimens of this taxon are known from the Lamar Member of the Bell Canyon Formation from the Guadalupe Mountains of west Texas, however, previously described specimens, now assigned to this taxon, indicate its stratigraphic range expands into the Roadian. The spines of this taxon are known from the Word Formation^23^ of the Glass Mountains, however, they were originally incorrectly described as secondary spines of a larger cidarid. These spines were collected from in between the Willis Ranch and Appel Ranch members of the Word Formation, which are lower Wordian in age^34^. Furthermore, Kier^24^ attributed a specimen from the Road Canyon Formation of the Glass Mountains to *Miocidaris* sp. This specimen (Figs 1A and 2D) is herein assigned to *Eotiaris guadalupensis*. This extends the stratigraphic range of this taxon into the Roadian, as the Road Canyon Formation is Roadian to Kungurian in age^34^,^36^,^37^. All of the material described herein has been silicified.

Morphologically, *E. guadalupensis* is very similar to *Eotiaris keyserlingi* from the Zechstein of the UK and Germany, differing significantly only in the morphology of its spines. Both bear rigidly sutured tests with plate imbrication adapically, sunken tubercles with multiple rows of scrobicular tubercles and crenulate and perforate tubercles. The spines of *E. keyserlingi,* which are well known^19^, are smooth and have much smaller spinules than those of *E. guadalupensis*^19^. They lack the clavate spine morphotype of *E. guadalupensis* and are much shorter. *E guadalupensis* also differs significantly from *E. connorsi*^24^. The test of *E. connorsi* is composed entirely of imbricate, non-rigid plates, while the tests of *E. guadalupensis* and *E. keyserlingi* are rigid except for adapically. The interambulacral plates in *E. connorsi* are also much wider and do not display densely packed scrobicular tubercles, as is the case in *E. guadalupensis* or *E. keyserlingi*.

*Occurrence—*Specimens are known from the Lamar Member of the Bell Canyon Formation of the Guadalupe Mountains, and the Road Canyon Formation and Word Formations of the Glass Mountains of west Texas. They are thus Roadian-Capitanian in age.

Localities are USNM 725e, 728p, and 738b from Cooper & Grant^38^ see supplementary information.

## Results

Phylogenetic analyses support the hypothesis that this taxon is a member of the cidaroidea (Fig. 2, Supplementary Figs S2, S3), and furthermore that it is sister taxon to *E. keyserlingi* (Supplementary Figs S2, S3; See Methods below). The euechinoid and cidaroid clades are confidently supported by bootstrap resampling (Supplementary Fig. S3) and *Eotiaris guadalupensis* is sister group to *E. keyserlingi* with a bootstrapped confidence interval of 83%. Because *Eotiaris guadalupensis* had apophyses and two columns of interambulacral plates, and plots as a cidaroid in the phylogenetic analyses (Supplementary Fig. S2, S3), then the strata from which it is known must be younger than the divergence time of euechinoids and cidaroids. Furthermore this provides a new basis upon which to obtain the hard minimum divergence date and thus is used to date the gene regulatory changes associated with this divergence. Following the best practices approach of Parham *et al.*^39^ a hard minimum divergence time was established for the divergence of the euechinoids and cidaroids. The oldest known occurrence of *Eotiaris guadalupensis* is the Road Canyon Formation of the Glass Mountains of west Texas. Based upon the presence of the transitional form between the conodonts *Jinogondolela idahoensis* and *J. nankingensis* and the presence of *J. nankingensis*, the Road Canyon Formation was determined to be Kungurian to Roadian in age^34^,^36^,^40^. Because the exact stratigraphic horizon within the Road Canyon Formation from which the specimen of *E. guadalupensis* was collected is unknown, the top of the Roadian stage was chosen as the hard minimum for the divergence of the cidaroids and euechinoids, following the conservative practices for establishing hard minima set forth by Parham *et al.*^39^. The top of the Roadian stage is set at 268.8 Ma based upon a smoothed cubic spline interpolation fit to the existing radiometric age dates for the Carboniferous and Permian,^41^ thus making the hard minimum divergence time for the euechinoids and cidaroids 268.8 Ma (Fig. 2). The discovery of this new taxon extends the minimum divergence time of the euechinoids and the cidaroids ten million years older than previously demonstrated^19^,^42^, shifting the minimum divergence time between these two taxonomic groups from Wuchiapingian (Lopingian) to Roadian (Guadalupian) (Fig. 2) and establishing that gene regulatory changes associated with this divergence must have also occurred by the Roadian.

## Discussion

The euechinoidea and cidaroidea are differentiated, in part, because of the structure of their Aristotle’s lanterns and perignathic girdles. The Aristotle’s Lantern operates as the “jaws” of the echinoid, and contains numerous calcareous elements including the teeth. The perignathic girdle comprises skeletal protrusions on the interior of the test that the retractor and protractor muscles, which move the lantern in and out of the test, attach to. Based upon the lantern and perignathic girdle structure of *Eotiaris keyserlingi*, Smith & Hollingworth^19^ determined that the euechinoids and cidaroids must have diverged prior to the Wuchiapingian stage (259.8 Ma). The perignathic girdle structures in the euechinoids and cidaroids are developmentally different, with the euechinoid auricles forming as protrusions from ambulacral plates and cidaroid apophyses developing from interambulacral plates^43^,^44^,^45^. Although euechinoids and cidaroids have differing perignathic girdle structures, neither structure is basal with respect to the other. This is known to be the case, because archaeocidarids, from which both the cidaroids and euechinoids likely evolved^3^,^19^, possessed the basal character state of having no perignathic girdle. *Eotiaris guadalupensis* also has two columns of interambulacral plates, and, through phylogenetic inference likely had two columns of ambulacral plates, as this character had been fixed in *Archaeocidaris* and its predecessors for approximately 90 Myr, since the Devonian^46^. These characters are synapomorphies of the crown group echinoids. As demonstrated in Fig. 2. and Supplementary Figures S2 and S3, the presence of apophyses, paired with two columns of interambulacral plates, indicates that *Eotiaris guadalupensis* is definitively a cidaroid, and thus the cidaroid lineage and euechinoid lineage must have already diverged prior to the appearance of this taxon in the rock record.

The presence of this taxon in Guadalupian rocks not only reinforces that the cidaroid-euechinoid divergence happened prior to the Permo-Triassic mass extinction^19^, but indicates that it had occurred by the Roadian (268.8 Ma; Fig. 2) at least 10 Myr earlier than previous estimates. Furthermore, the potential exists for new discoveries to show that it may be even earlier, especially given that *Eotiaris guadalupensis* does not plot as the most basal cidaroid in the phylogenetic analyses (Supplementary Fig. S2). In addition, this indicates that crown-group echinoids may have been established by the Guadalupian and were certainly biogeographically widespread by the Lopingian^24^. The appearance of *Eotiaris guadalupensis* in the Roadian also extends the inferred range of euechinoids prior to the Permian-Triassic boundary. The oldest definitive euechinoids, *Hemipedina hudsoni* and *Diademopsis heberti* are not known until the Norian (Late Triassic)^47^,^48^,^49^ thus making the implied fossil gap a minimum of 40 Myr. This new species also likely has profound impacts on the molecular clock divergence dating for all echinoid clades. As the divergence of the cidaroids and euechinoids is the root divergence node used for all divergence-dating analyses of echinoids^42^,^50^, this new taxon has pushed back the basal node for divergence analyses 10 Myr. Future work will attempt to incorporate this new basal divergence node into molecular clock analyses.

Underlying this phylogenetic divergence must have been large-scale reorganization of the developmental GRNs of cidaroids and euechinoids, with profound impacts on the differential development of these clades. With regard to post-larval development, *E. guadalupensis* and other basal stem-group cidaroids are morphologically very similar to even the most derived members of the crown group cidaroidea, due to the conserved nature of the cidaroid body plan. Developmentally, this poses an interesting comparison with the euechinoidea, which have a much higher degree of post-larval morphological disparity relative to the cidaroids^1^,^2^. New evidence has also shed light on the gene regulatory development of juvenile skeletal structures, particularly with regard to the development of apophyses and auricles. Both apophyses and auricles develop through the expression of specific genes known to be required for skeletogenic specification in embryonic and post-embryonic development: *sm37, alx1*, and *vegfR*^45^. In particular, *sm37* is a well-understood biomineralization gene^51^,^52^ the expression of which is regulated by the upstream transcription factor *alx1*^6^,^53^. The differential spatial deployment of these genes during skeletogenesis is controlled by *vegfR* in the embryo^54^, and as such, this gene may be responsible for the differential spatial expression of *alx1* and *sm37* during the formation of apophyses and auricles^45^. Because of the presence of *Eotiaris guadalupensis*, which has definite apophyses, in the Roadian, the fixation of the differential deployment of these biomineralization genes must have at least begun by 268.8 Ma.

Additionally, there are a number of larval and embryonic developmental differences between modern cidaroids and euechinoids that must have arisen with the divergence of these two clades in the Permian. Euechinoid embryos possess four micromeres, and their larval skeleton arises from primary mesenchymal cells, which ingress at the vegetal pole of the embryo^10^. Cidaroids, however, have a variable number of micromeres^8^,^9^,^55^ and lack primary mesenchymal cells, instead deriving their larval skeleton from skeletogenic cells emerging along with other mesodermal cells from the tip of the archenteron^8^,^9^,^56^. In euechinoids, the specification of skeletogenic mesenchyme is regulated by the double-negative gate, whereby in the micromere lineage, *pmar1* represses *hesC*, which then allows for the expression of downstream genes responsible for micromere specification such as *alx1, ets1,* and *tbr*^6^,^57^. The double negative gate appears to be responsible for skeletogenic micromere specification across numerous phylogenetically diverged euechinoid lineages, including the stomopneustoids, spatangoids, clypeasteroids and camaradonts^58^ such that it is very likely present throughout all indirect developing euechinoids. Contrary to euechinoids, it has been demonstrated that cidaroids lack the *hesC* mediated double negative gate^7^ and that *tbr* plays no role in skeletogenesis^7^. Many of the genes encoding transcription factors and biomineralization genes responsible for micromere specification and embryonic skeletogenesis in euechinoids are also involved in juvenile euechinoid skeletogenesis and were likely co-opted by the skeletogenic micromere lineage^59^. As the euechinoids alone possess a larval skeleton that is derived from primary mesenchymal cells, it is likely that this co-option of juvenile skeletogenic genes occurred with the divergence of cidaroids and euechinoids. It is unknown as to whether the euechinoid or cidaroid suites are ancestral, however, this new fossil evidence indicates that the acquisition of one of these two differential character suites must have occurred since the divergence of the euechinoids and cidaroids in the Roadian (268.8 Ma) and is potentially very ancient.

## Conclusions

*Eotiaris guadalupensis*, the geologically oldest cidaroid, is the oldest known probable crown-group echinoid in the fossil record. This taxon pushes back the divergence of the crown-group echinoids, the cidaroids and the euechinoids, to at least 268.8 Ma in the Roadian stage of the Permian. It furthermore extends the inferred range of early euechinoids and establishes a new hard minimum divergence for the basal node of all divergence dating studies regarding the echinoidea. In light of recent discoveries of differential cidaroid and euechinoid embryonic and juvenile development, this taxon also provides strong evidence for fixation of disparate gene expression systems by the Roadian. *Eotiaris guadalupensis* provides direct evidence for the differential spatial expression of specific genes in euechinoid and cidaroid post-metamorphosis skeletogenesis and indicates that this differential spatial expression must have been established by at least 268.8 million years ago.

## Methods

Specimens of *Eotiaris guadalupensis* were analysed using dissecting microscopes and ESEM microscopy was used to determine mineralogy of specimens. Measurements were taken with calipers. Phylogenetic analyses were undertaken to rigorously demonstrate the phylogenetic relationships of this species with respect to other Permian and Triassic echinoids. Permian and Triassic euechinoids (three species; all from the family Pedinidae) and cidaroids (three species; two from the family Miocidaridae and one from the Triadotiaridae) were included in the analysis, in addition to *E. guadalupensis*. The outgroup of the analysis was *Archaeocidaris whatleyensis*, a well-known, stem-group echinoid, which has been used as outgroup to all crown group echinoids in previous analyses^1^,^2^,^49^. The characters used in the phylogenetic analysis in Supplementary Figures S2 and S3 consisted of 24 characters, 20 were binary and 4 were multistate. Characters and character states are in supplementary information. All characters were unordered and unweighted in original analyses and character matrix is listed in Supplementary Table S1. Corresponding Nexus file is in supplementary information. Initial phylogenetic analysis was run in PAUP version 4^60^ and consisted of an exhaustive search of all possible trees. This analysis resulted in 2 most parsimonious trees with length 31 consistency index (CI) .806 and retention index (RI) .750. Characters were then reweighted by their maximum retention indices and analyses were rerun. This resulted in one most parsimonious tree, equal to one of the two resultant trees from the unweighted search and with length 22.5, CI .911 and RI .875 (Supplementary Fig. S2). In order to estimate branch support we ran a heuristic search with 1000 RASs and TBR with 1000 bootstrap replicates on the reweighted character matrix. Bootstrapped confidence intervals are shown with appropriate branches in Supplementary Figure S3.

## Additional Information

**How to cite this article**: Thompson, J. R. *et al.* Reorganization of sea urchin gene regulatory networks at least 268 million years ago as revealed by oldest fossil cidaroid echinoid. *Sci. Rep.* **5**, 15541; doi: 10.1038/srep15541 (2015).

## Supplementary Material

Supplementary Information
