# Supplementary material for: Reorganization of sea urchin gene regulatory networks at least 268 million years ago as revealed by oldest fossil cidaroid echinoid
Source: Sci Rep. 2015 Oct 21;5:15541. doi: 10.1038/srep15541 (PMC4614444; doi:10.1038/srep15541)
Supplement: Supplementary Information [file srep15541-s1.pdf]

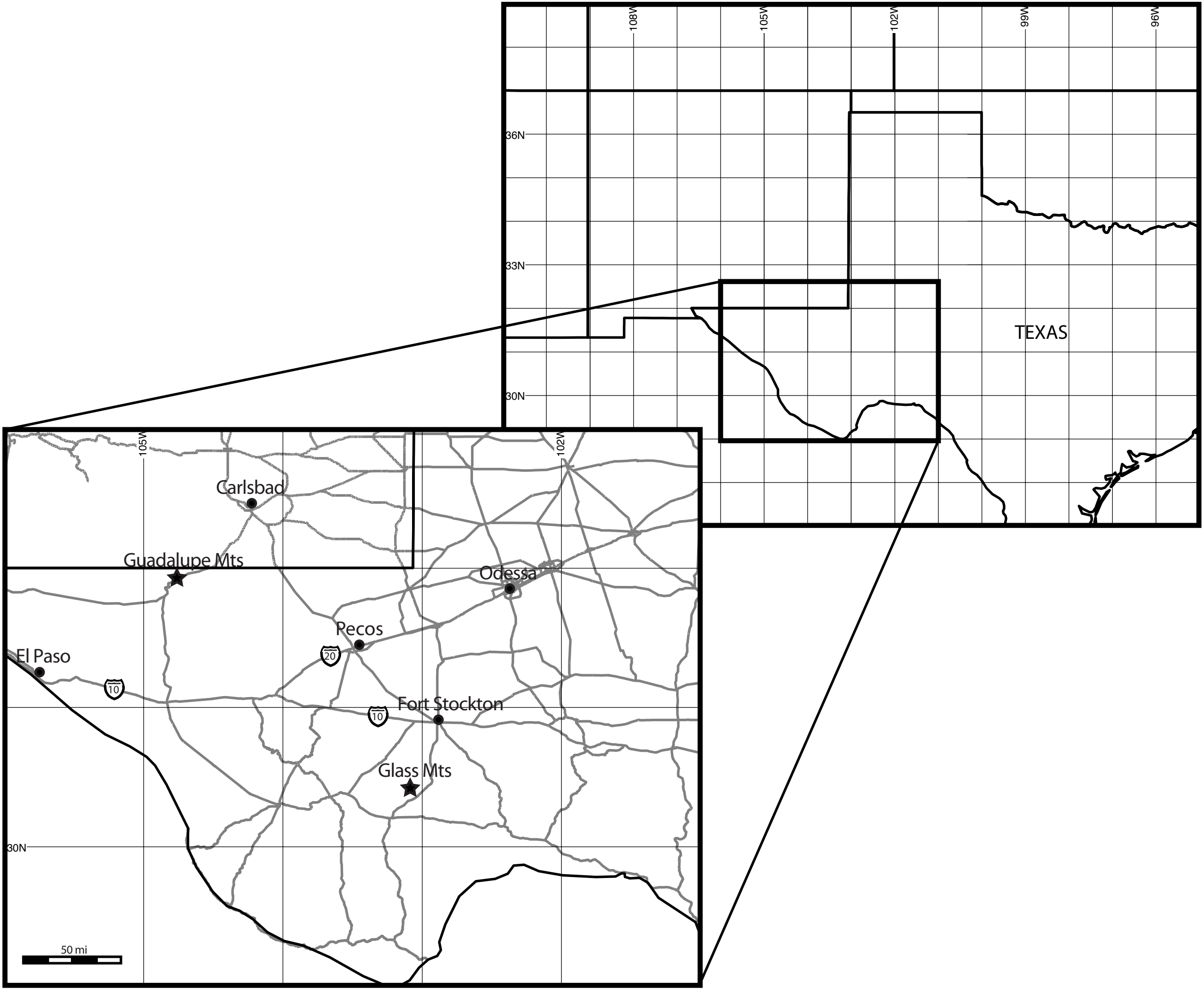

Supplementary Figure S1.

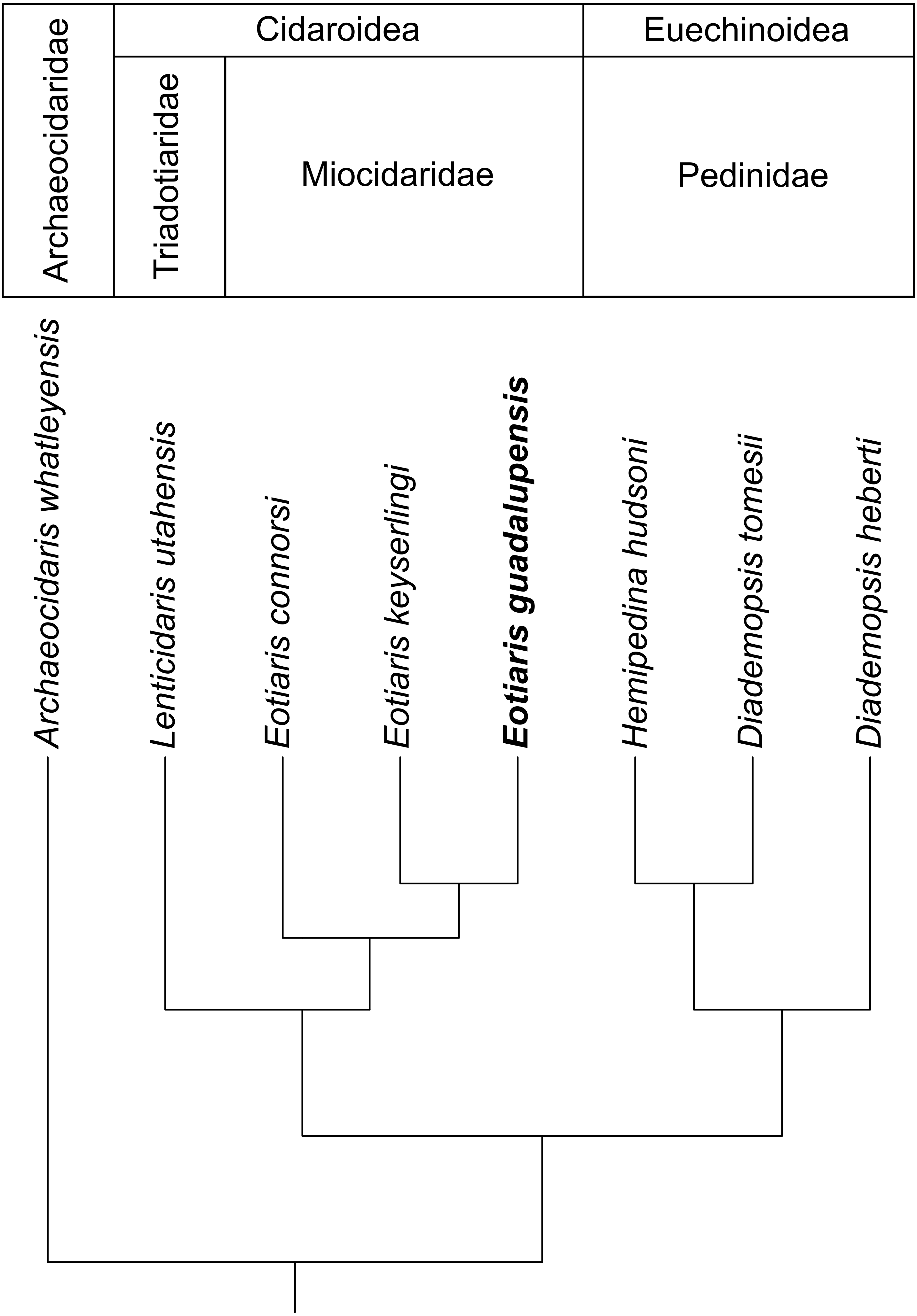

Supplementary Figure S2.

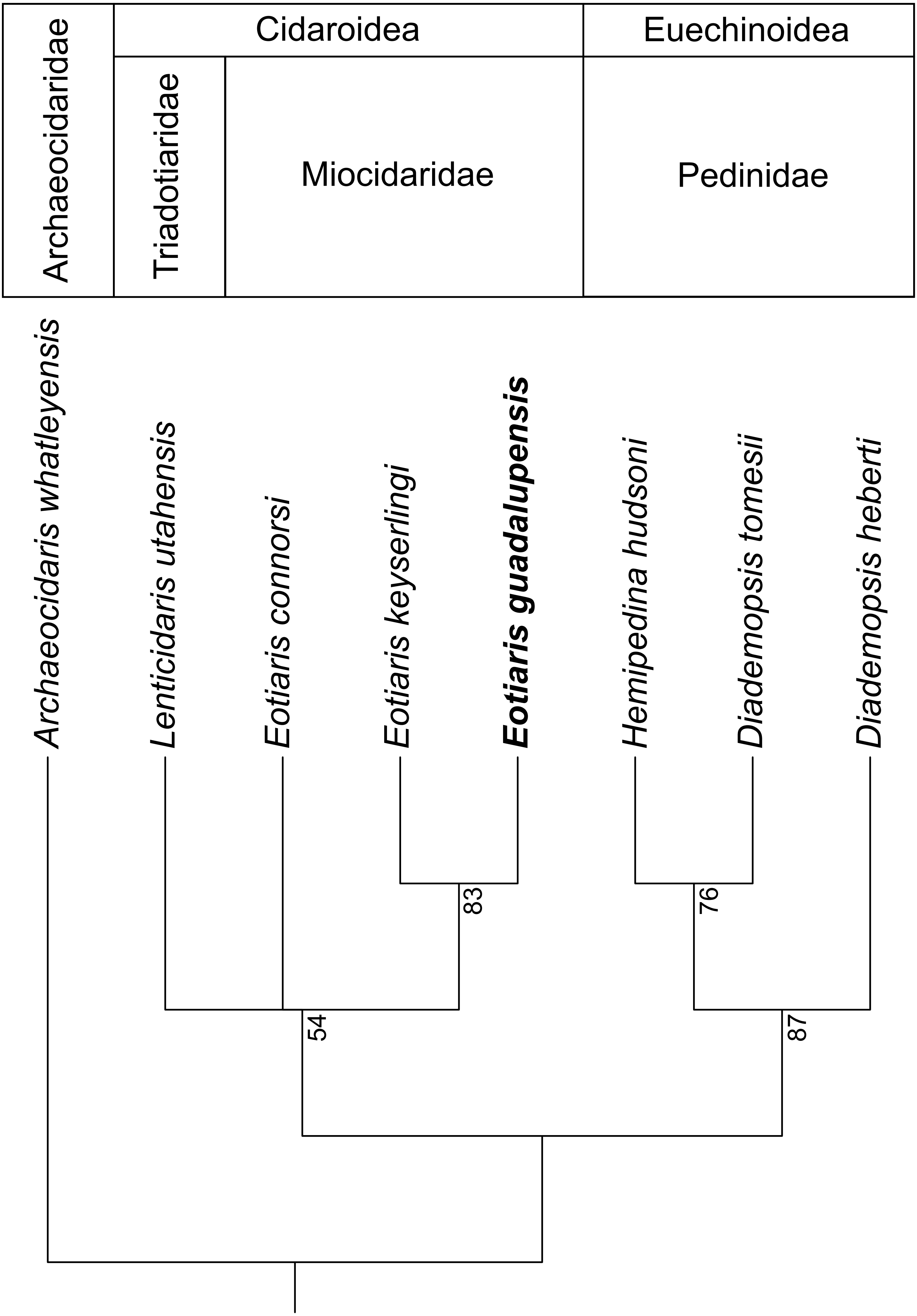

Supplementary Figure S3.

Supplementary Table S1

Character Number	#1	#2	#3	#4	#5	#6	#7	#8	#9	#10	#11	#12	#13	#14	#15	#16	#17	#18	#19	#20	#21	#22	#23	#24
Archaeocidaris whatlyensis	1	0	0	0	0	0	1	0	0	0	0	0	0	0	1	0	0	0	0	0	0	1	1	0
Lenticidaris utahensis	1	1	1	0	1	1	0	0	1	1	0	2	0	0	1	0	0	0	0	0	0	1	0	0
Eotiaris connorsi	1	1	1	?	1	1	?	0	1	1	0	2	0	0	1	0	1	0	0	0	0	1	1	0
Eotiaris keyserlingi	0	1	1	1	1	1	0	0	0	1	1	2	0	0	?	?	1	?	?	?	?	1	1	0
Eotiaris guadalupensis	0	1	1	1	1	?	?	0	0	1	1	2	0	0	?	?	1	?	?	?	?	1	1	1
Hemipedina hudsoni	0	1	1	2	2	?	?	1	0	?	?	?	0	0	1	1	2	0	?	1	1	?	?	?
Diademopsis heberti	0	0	1	2	?	?	?	?	1	0	0	1	0	1	1	0	2	0	1	1	0	?	?	?
Diademopsis tomesii	1	1	1	2	2	?	?	1	1	0	0	1	0	1	1	1	2	0	1	1	1	0	?	?

Reorganization of sea urchin gene regulatory networks at least 268 million years ago as revealed by oldest fossil cidaroid echinoid

Jeffrey R. Thompson[1*], Elizabeth Petsios[1], Eric, H. Davidson[2], Eric M. Erkenbrack[2], Feng Gao[2], and David J. Bottjer[1]
[1]Department of Earth Sciences, University of Southern California, Los Angeles, California 90089-0740
[2]Division of Biology, California Institute of Technology, Pasadena, California, 91125
*thompsjr@usc.edu

Supplementary Information

Geological Setting

Based on the concurrent presence of the conodonts *Jinogondolella postserrata* and *J. shannoni*, the Lamar Member of the Bell Canyon Formation has been determined to be Capitanian (Guadalupian) in age[1,2]. The transition from the *J. postserrata* zone to the *J. shannoni* zone takes place within the uppermost Lamar Limestone Member[3] thus clarifying the age to lower Capitanian (~264 Ma)[4]. The uppermost Lamar Limestone Member also marks the transition from *J. shannoni* to *J. altudaensis*. The presence of both of these faunal transitions clarifies the age of the Lamar Limestone Member to Lower Capitanian (264-263 Ma).

The Lamar Member of the Bell Canyon Formation is a wedge-shaped carbonate unit that was deposited in the Delaware Basin adjacent to the Guadalupe Mountains and the Capitan Formation to the Northwest. This deposit contains carbonate debris flows transported from the reef edge of the Capitan Formation. It is over 90 m thick near the shelf margin, where allochthonous sedimentation was greatest, and thins basinward to only about 2 m in thickness[5]. The Capitan Formation and the Bell Canyon Formation are correlable[3], and the two formations merge towards the edge of the Delaware basin. Lambert *et al.*[3] describe the Lamar Limestone Member as containing medium to dark grey organic-rich mudstones, and skeletal, peloidal carbonate wackestones and packstones with carbonate debris flows containing silicified fossils. This is at the type section of the Reef Trail Member (overlying the Lamar Limestone Member) and is fairly close to the basin edge[3]. Babcock[5] noted numerous transported silicified reef fossils infilling channels in the zone proximal to the reef. Proximal to the reef edge, the fauna of the Lamar Limestone Member consists of common brachiopods, bryozoans and crinoids[5]. Cooper and Grant[6] noted that the brachiopod fauna in the Lamar Limestone was similar to that occurring on the 'reef slope'. Additionally, according to Babcock[5] (Pp. 365, Fig. 5) silicified echinoid

spines and plates are common in these debris flows. Basinward, the Lamar Limestone Member thins, and is composed primarily of finely laminated mud lacking fossils and bioturbation[5]. The specimens were collected from localities USNM 725e, 728p, and 738b near the Guadalupe Mountains[6] and thus were collected from near the shelf margin.

Previously oldest cidaroids

Previous to this study, the earliest known definitive cidaroid was *Eotiaris keyserlingi*[7]. *Eotiaris keyserlingi* is known from the Ford Formation of the Zechstein Cycle 1 of the Sunderland District, U.K and from the Zechstein of Germany[8]. Though biostratigraphic correlation of the Zechstein with other Permian rocks worldwide has been difficult[4] based upon conodont biostratigraphy, the Zechstein has been determined to be Wuchiapingian[9]. Additionally, recent $\delta^{13}C$ correlation has shown that the Zechstein in Poland, which based upon the presence of the conodont *Millerina divergens* is correlative with the English Zechstein[10,11], was likely deposited after the Wuchiapingian Isotope Event 0 which makes its age approximately 258-257 Ma[12]. Even by considering the more conservative ages of 268.8 Ma and 258 Ma for *E. guadalupensis* and *E. keyserlingi* respectively, the discovery of this new taxon extends the minimum divergence time of the euechinoids and the cidaroids ten million years older than previously thought[7,13], shifting the minimum divergence time between these two taxonomic groups from Wuchiapingian (Lopingian) to Roadian (Guadalupian). The age of *E. keyserlingi* has incorrectly been reported with age ranges for the taxon assigned to the Roadian[14] and Kungurian[15], however because it is known only from the Zechstein, which is confidently assigned to the Wuchiapingian[9-11], *E. keyserlingi* is Wuchiapingian, and thus Upper Permian, in age.

Specimen localities

Localities from which these specimens were collected are from Cooper & Grant[6]. Latitude and longitude coordinates for these localities were further determined by Wardlaw[16]. USNM 725e is located at 31.9474, 104.7075, USNM 728p is at 31.942, 104.701, and USNM 738b at 31.981, 104.7497..

Specimens described by Kier[17,18] from Cooper and Grant's localities USNM 706b and 716x. USNM 706b is described as a thin lens between the Willis Ranch and Appel Ranch members of the Word Formation 0.2 miles southwest of junction of Hess Canyon with its south branch, 1.9 miles north 65° west of Old Word Ranch site, and 4.78 miles north 34.5° east of Hess Ranch house, Hess Canyon quadrangle. USNM 716x is 1.5 miles north 20° west of Hess Ranch, 1.03 miles north 63° east of hill 5801, Hess Canyon quadrangle. Both of these localities were originally regarded as Wordian, however, Cooper & Grant[19] updated the age of 716x to Roadian.

Characters and character states for phylogenetic analysis

Test Characters

1. (0) 9 or more interambulacral plates per column; (1) Less than 9 interambulacral plates per column
2. Width of interradial extrascrobicular area is (0) <1/3 plate width (1) >1/3 plate width
3. (0) Interambulacral zone composed of more than 2 columns of plates (1) Interambulacral zone has 2 columns of plates
4. (0) Plates imbricate throughout test; (1) Plates imbricate adapically only; (2) Test rigid

Oral Characters

5. (0) No perignathic girdle; (1) Perignathic girdle composed of apophyses; (2) Perignathic girdle composed of auricles
6. Tooth cross sectional shape (0) Flat; (1) U-shaped
7. Serration of tooth tip (0) Absent; (1) Present
8. Buccal notches (0) Absent; (1) Present

Interambulacral Characters

9. Interambulacral plate width to height dimensions (0) <2; (1) >2
10. Tubercle crenulation (0) Absent (1) Present
11. Boss undercut (0) No (1) Yes
12. Areoles confluent (0) No (1) Yes (whole test) (2) Yes (orally only)
13. Well organized scrobicular ring (relative to irregularly distributed granules) (0) No (1) Yes
14. (0) Single primary tubercle per interambulacral plate at ambitus (1) Multiple large tubercles per interambulacral plate at ambitus

Ambulacral Characters

15. All ambulacral plates in contact with perradius (0) No (1) Yes
16. Pore pairs uniserial (0) biserial (1)
17. Columns (0) imbricate interradially at ambitus (1) imbricate adradially at ambitus (2) are rigid at ambitus
18. Ambulacral plating pseudocompound (0) No (1) yes (adorally only) (2) Yes (whole test)
19. Primary ambulacral tubercles perforate (0) No (1) Yes
20. Ambulacral demiplates fused by tubercle overgrowth (0) No (simple plating) (1) Yes (compound plating)
21. (0) Distinct Primary tubercle on ambulacral plates or (1) multiple granules

Spine Characters

22. Spine hollow (0) No (1) Yes
23. Spine sculpturing (0) Smooth (1) with spinules
24. Spine shape (0) Straight (1) Clavate

Supplemental References

1. Lambert, L. L., Wardlaw, B. R., Nestell, M. K., & Nestell, G. P. Latest Guadalupian (Middle Permian) conodonts and foraminifers from West Texas. *Micropaleontology* **48**, 343-364 (2002).

2. Lambert, L. L. in *Basinal Facies of the Uppermost Guadalupian: Applicability to Exploration and Development Projects; Field Trip Guidebook Permian Basin Section, SEPM Publication 2006-46*. (ed. Hinterlong, G) 78-85 (PBS-SEPM, 2006).

3. Lambert, L. L., Bell J. R., G. L., Fronimos, J. A., Wardlaw, B. R., & Yisa, M. O. Conodont biostratigraphy of a more complete Reef Trail Member section near the type section, latest Guadalupian Series type region. *Micropaleontology* **56**, 233-256 (2010).

4. Henderson, C. M., Davyvov, V. I., & Wardlaw, B. R. in *The Geologic Timescale 2012* (eds Gradstein, F., Ogg, J., Schmitz, M. & Ogg, G.) 653-680 (Elsevier, Amsterdam 2012).

5. Babcock, L. C. in *Upper Guadalupian facies, Permian reef complex, Guadalupe Mountains, New Mexico and west Texas: Permian Basin Section SEPM Publication* (eds Hileman, M. & S. J. Mazzullo, S. J.) 357-389 (SEPM, 1997)

6. Cooper, A. G., & Grant, R. E. Permian Brachiopods of West Texas, Part I. *Smithson. Contrib. Paleobiol.* **14**, (1972).

7. Smith, A. B., & Hollingworth, N. T. J. Tooth structure and phylogeny of the Upper Permian echinoid *Miocidaris keyserlingi*. *P. Yorks. Geol. Soc.* **48**, 47-60 (1990).

8. Reich, M. *Linguaserra spandeli* sp. nov. (Echinodermata: Ophiocistioidea) from the Late Permian (Zechstein) of Thuringia, Germany. *Ann. Paléontol.* **93**, 317-330 (2007).

9. Mei, S. & Henderson, C. M. Evolution of Permian conodont provincialism and its significance in global correlation and paleoclimate implication. *Palaeogeogr. Palaeoclimatol. Palaeoecol.* **170**, 237-260 (2001).

10. Szaniawski, H. Conodonts of the Upper Permian of Poland. *Acta Palaeontol. Pol.* **69**, 325-342 (1969).

11. Swift, A. The conodont *Merrillina divergens* (Bender & Stoppel) from the Upper Permian of England. *Geol. Soc., London, Spec. Publ.* **22**, 55-62 (1986).

12. Peryt, T. M., Durakiewicz, T., Kotarba, M. J., Oszczepalski, S., & Peryt, D. Carbon isotope stratigraphy of the basal Zechstein (Lopingian) strata in Northern Poland and its global correlation. *Geol. Q.* **56**, 285-298 (2012).

13. Smith, A. B., Pisani, D., Mackenzie-Dodds, J. A., Stockley, B., Webster, B. L., & Littlewood, T. J**.** Testing the Molecular Clock: Molecular and Paleontological Estimates of Divergence Times in the Echinoidea (Echinodermata). *Mol. Biol. Evol.* **23**, 1832-1851 (2006).

14. Nowak, M. D., Smith, A. B., Simpson, C., & Zwickl, D. J. A simple method for estimating informative node age priors for the fossil calibration of molecular divergence time analyses. *PLoS One* **8**, e66245 (2013).

15. Hopkins, M. J. & Smith, A. B. Dynamic evolutionary change in post-Palaeozoic echinoids and the importance of scale when interpreting changes in rages of evolution. *Proc. Natl. Acad. Sci. U.S.A.* **112,** 3758-3763 (2015).

16. Wardlaw, B. R. Paleontologic database for the Guadalupe Peak 1:100,000 Quadrangle: A prototype for the National Paleontologic Database, Paleodata. *U.S. Geological Survey Open-File Report 2008-1141* [99] (2008).

17. Kier, P. M. Permian echinoids from West Texas. *J. Paleo.* **32**, 889-892 (1958).

18. Kier, P. M. Evolutionary trends in Paleozoic echinoids. *J. Paleo*. **39**, 436-465 (1965).

19. Cooper, A. G., & Grant, R. E. Permian Rock Units in the Glass Mountains West Texas. *U. S. Geol. Surv. Bull.* **1244-E**, E1-E9 (1966).

20. Amante, C. & Eakins, B.W. ETOPO1 Arc-Minute Global Relief Model: Procedures, Data Sources and Analysis. NOAA Technical Memorandum NESDIS DGDC-24. National Geophysical Data Center, NOAA. (2009).

21. Federal Highway Administration. National Highway Planning Network edition 14.5. (2014).

Supplementary Figure Captions

Supplemental Figure S1. Map detailing the localities from which the specimens were collected. Stars indicate generalized locality positions. Map made using Grass GIS v7.1 using ETOPO1 elevation data[20] with state boundaries made with Natural Earth. Free vector and raster map data @ naturalearthdata.com. Highways from the Federal Highway Administration[21].

Supplemental Figure S2. Cladogram displaying single most parsimonious tree from a single reweighting of characters by their retention indices representing phylogenetic relationships between *Eotiaris guadalupensis* n sp. and other Triassic euechinoids and cidaroids.

Supplemental Figure S3. Cladogram showing relationships of *Eotiaris guadalupensis* n sp. to other Triassic euechinoids and cidaroids. Numbers represent bootstrapped confidence intervals from a heuristic search using 1000 bootstrap replicates with 1000 RASs and TBR.

Supplemental Table 1. Character matrix for phylogenetic analysis.
